# Supplementary material for: The ambiguity of nestedness under soft and hard constraints
Source: Sci Rep. 2020 Nov 16;10:19903. doi: 10.1038/s41598-020-76300-1 (PMC7669898; doi:10.1038/s41598-020-76300-1)
Supplement: Supplementary file 1 — Supplementary Information 1. [file 41598_2020_76300_MOESM1_ESM.pdf]

The impact of statistical ensembles non-equivalence on nestedness measurements
Supplementary Information

Matteo Bruno[1,*], **Fabio Saracco**[1], **Diego Garlaschelli**[1,2], **Claudio J. Tessone**[3], **and Guido Caldarelli**[1,4,5,6]

[1]IMT School for Advanced Studies, P.zza S. Francesco 19, 55100 Lucca (Italy)
[2]Lorentz Institute for Theoretical Physics, University of Leiden, Niels Bohrweg 2, 2333 CA Leiden (The Netherlands)
[3]URPP Social Networks, University of Zürich, Andreasstrasse 15, CH-8050 Zürich (Switzerland)
[4]European Centre for Living Technology, Università di Venezia “Ca’ Foscari”, S. Marco 2940, 30124 Venice (Italy)
[5]Catchy srl, Talent Garden Poste Italiane, Via Giuseppe Andreoli 9, 00195 Rome (Italy)
[6]Istituto dei Sistemi Complessi CNR, Dip. Fisica, Università Sapienza, P.le Aldo Moro 2, 00185 Rome (Italy)
[*]matteo.bruno@imtlucca.it

1 sNODF vs. SNES

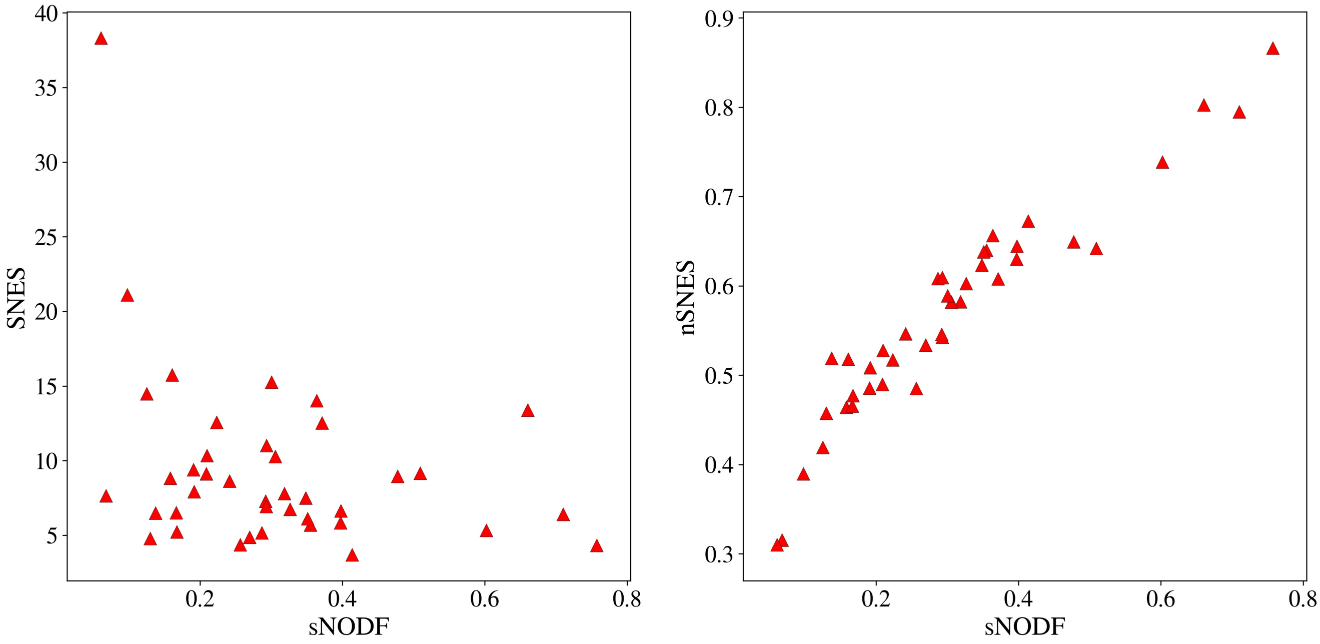

Figure A. sNODF vs SNES (left) and vs nSNES (right) for the 40 networks of the Bascompte dataset. Spearman correlation coefficients are, respectively, -0.26 and 0.96. The figure is very similar to Fig. 1 in the main text, so we included only one in it.

In the main text we showed the correlation between the NODF and the SNES, in its two different normalizations. In Fig. A it is possible to observe that an analogous relation is present between the sNODF and the SNES measures.

2 Perfectly Nested Networks

For a PNN, the corresponding ensembles (both microcanonical and canonical) are singular, i.e. the only matrix in the ensembles is the PNN itself. In this section we explain this fact.
Let us start from the microcanonical ensemble. First, let us summarise the main steps of the Curveball:

1. Select at random a couple of nodes on the same layer (for making the example clearer let us consider, in full generality, $i,\ j \in N_{\mathrm{L}}$);

2. Check that the neighbourhoods of the nodes are not perfectly overlapping: if so, start again.

3. Take the set of uncommon neighbours $U(i,j) = \{\alpha \in N_\Gamma | (m_{i\alpha} = 0 \&\& m_{j\alpha} = 1) || (m_{i\alpha} = 1 \&\& m_{j\alpha} = 0)\}$ and remove them from the neighbourhood of both;

4. Assign $k_i - \sum_\alpha m_{i\alpha} m_{j\alpha}$ new neighbours to node i, chosen at random from $U(i,j)$ and the rest of the nodes in $U(i,j)$ to node j.

Consider the case in which $k_i = k_j$: due to PNN nature, $U(i,j) = \emptyset$ and the algorithm stops at the step 2. Then, consider the case $k_i > k_j$: $U(i,j)$ contains only the connections that i has and j has not (due to the perfect nestedness of the network, all connections of j are connections of i too). Then, at step 4, the number of new neighbours of j is $k_j - \sum_\alpha m_{i\alpha} m_{j\alpha} = 0$, while the same quantity is exactly $|U(i,j)|$ for i, thus the algorithm is stuck in the present configuration. A similar intuition can be found in[1].

In the canonical ensemble the situation is a little more involved. Let us consider, as an example, the biadjacency matrix in Fig. 2 in the main text, representing a PNN; the presented arguments can be generalised to any PNN. Due to the ordering we imposed on the biadjacency, if rows and columns represent respectively the L and the Γ layers, we have:

$$\begin{aligned} \langle k_1 \rangle &= \sum_{\alpha}^{N_\Gamma} p_{1\alpha} = k_1^* = N_\Gamma; \\ \langle h_1 \rangle &= \sum_{i}^{N_\mathrm{L}} p_{i1} = h_1^* = N_\mathrm{L}, \end{aligned} \tag{1}$$

which can be satisfied if and only if $p_{1\alpha} = 1, \forall \alpha \in \Gamma$ and $p_{i1} = 1, \forall i \in \mathrm{L}$. Thus all entries involving the fully connected nodes are deterministic. Such a conclusion has implications, on the opposite side of the biadjacency matrix:

$$\begin{aligned} \langle k_{N_\mathrm{L}} \rangle &= \sum_{\alpha>1}^{N_\Gamma} p_{N_\mathrm{L}\alpha} + 1 = k_{N_\mathrm{L}}^* = 1; \\ \langle h_{N_\Gamma} \rangle &= \sum_{i>1}^{N_\mathrm{L}} p_{iN_\Gamma} + 1 = h_{N_\Gamma}^* = 1, \end{aligned} \tag{2}$$

which, in turns, implies $p_{N_\mathrm{L}\alpha} = 0, \forall \alpha > 1 \in \Gamma$ and $p_{iN_\Gamma} = 0, \forall i > 1 \in \mathrm{L}$, i.e. the entries of nodes with only a single connection are deterministic too. Then let us pass to consider again the first nodes:

$$\begin{aligned} \langle k_2 \rangle &= 1 + \sum_{\alpha>1}^{N_\Gamma - 1} p_{2\alpha} + 0 = k_2^* = N_\Gamma - 1; \\ \langle h_2 \rangle = \langle h_3 \rangle &= 1 + \sum_{i>1}^{N_\mathrm{L}-1} p_{i2} + 0 = 1 + \sum_{i>1}^{N_\mathrm{L}-1} p_{i3} + 0 = h_2^* = h_3^* = N_\mathrm{L} - 1 \end{aligned} \tag{3}$$

(in the second line we use the fact that columns 2 and 3 have the same degree, thus their Lagrangian multipliers are equal and so $p_{i2} = p_{i3}, \forall i \in \mathrm{L}$). Let us first focus on equation 3: we have $N_\Gamma - 2$ unknown probabilities, summing to $N_\Gamma - 2$. Thus $p_{2\alpha} = 1$ for $1 < \alpha < N_\Gamma$. Analogous considerations are valid for all p_{i2}s and p_{i3}s and thus these entries are again deterministic. Iteratively discounting the information obtained at the previous steps, it is possible to show that the canonical ensemble of a PNN is composed by a single graph, or, more correctly, the probability for every representative in the ensemble is 0 but for the PNN itself (which, instead has $P(PNN) = 1$).

3 Isolated nodes in the canonical ensemble

In the canonical ensemble, the degree sequence is fixed on average, thus there are fluctuations from realisation to realisation. Therefore, nodes with low degree, say close to 1, in the real network, can result as isolated in some realisations of the ensemble. As it can be seen from Fig. B, the average of isolated nodes over the size of the network is nearly constant all over the dataset.

4 SNES dependence on the number of nodes

In the paragraph III.C.2 in the main text, we observed on the canonical samples that spectral radius is a little greater than the true value. Our intuition is that on average, out of two matrices with the same number of links, the one with the smallest number of nodes has the largest radius. Fig. C shows a little experiment confirming our guess: we generate synthetic networks of various sizes, but with the same number of links. As it can be seen, as the size increases, the average nSNES reduces. Something similar happens for the sNODF.

5 Canonical vs microcanonical ensemble: more examples of ensembles distributions

In Fig. 5 in the main text we showed the realizations of the canonical and microcanonical ensembles. In Fig. D we show the same plot for two more real networks. While the observed values are not as extreme as the one presented in the Fig. 5 of the main text, still the same behaviours are present: a positive correlation between the nSNES and sNODF in the canonical ensemble and a negative one in the microcanonical case. Let us remark that the observed values of both nSNES and (heterogeneous) sNODF represent the negative extremes in the case of the canonical ensemble in the dataset 5 (left panel of Fig. D), while they fall almost in the center of the distribution of the microcanonical ensemble. Even more striking is the case of the dataset 1 (right panel of Fig. D): while the observed (heterogeneous) sNODF is extremely low both for the canonical and microcanonical ensembles, the observed nSNES is much greater than expected in the microcanonical ensemble, while it stays in the middle of the distribution in the canonical one. As already mentioned in the main text, the value of the homogeneous and heterogeneous sNODF have opposite behaviours in the canonical ensemble, as it can be seen in both panels of Fig. D.

6 Assortativity vs. nestedness in the microcanonical and canonical ensemble

In the paragraph III.C.3 in the main text, we observed a high correlation between nSNES and the assortativity of the sampled networks, and an anticorrelation of both with the sNODF in the microcanonical ensemble. In Fig. 7 of the main text, in the panels on the left, we show that this is not as evident on the measurements of our dataset without filtering and both nSNES and sNODF show a weak anticorrelation with the assortativity. When, instead, the measures on the real data are compared with the microcanonical ensemble, a strong correlation is evident. In particular, the z-scores of the sNODF (SNES) anticorrelate (correlate) with the z-scores of assortativity, showing different behaviours for the two metrics, see the two panels on the right of Fig. 7 of the main text.

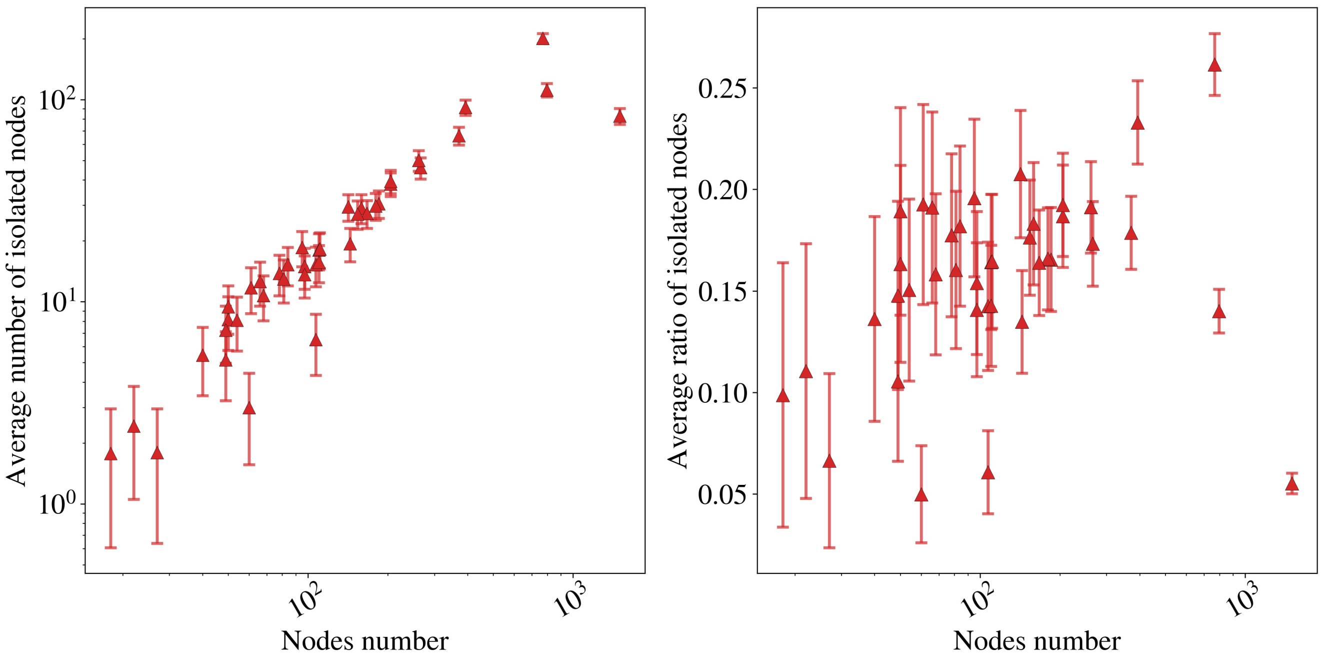

Figure B. The average number (left) and ratio (right) of isolated nodes in the canonical ensemble samples as a function of the total nodes, with error bars representing one standard deviation. The relative Spearman correlation coefficients are 0.96 (left) and 0.36 (right).

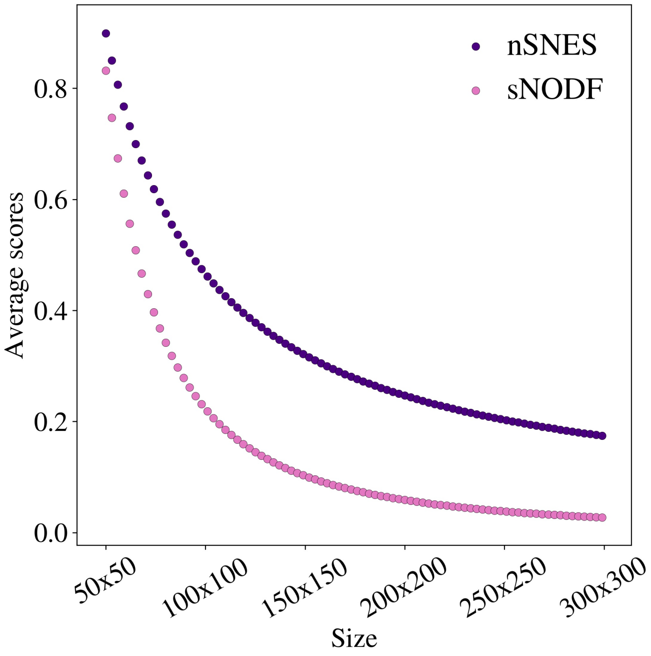

Figure C. In this experiment we generate random bipartite networks of various sizes, filling them with exactly 2000 links in random positions. For every size considered, 1000 samples have been generated and we measured the resulting average nSNES and sNODF. We omit the corresponding standard deviations because they are negligible. Although this is not a rigorous argument since many of the considered networks could have isolated nodes, it indicates that reduced average dimension with fixed average links number can generate a bias in the nSNES and NODF/sNODF measures in the canonical ensemble.

In the canonical ensemble instead, the correlations that we find in the microcanonical ensemble are almost completely lost, as it can be seen in Fig. F. Here the fluctuations cover completely the relations between the assortativity and the various nestedness metrics.

Another example of the behaviour of assortativity in the canonical ensemble is provided in Fig. E, in which we present the analogous of Fig. 6 in the main text for the canonical ensemble. As it can be seen from the second and third panels, almost no correlation between the assortativity and the various nestedness measures can be observed, while the correlation between the two nestedness metrics is evident in the first panel. Also in this case, the overestimation of the measures in the canonical ensemble screens the anticorrelation observed in Fig. 6.

7 Homogeneous vs microcanonical sNODF

Although the homogeneous sNODF seems correlated with the microcanonical one by looking at Fig. 3 in the main text, quantifying their differences is not an easy task. In Fig. G we show that the difference in the respective z-scores seems to be uncorrelated with the average number of isolated nodes in a network sampled from the canonical ensemble.

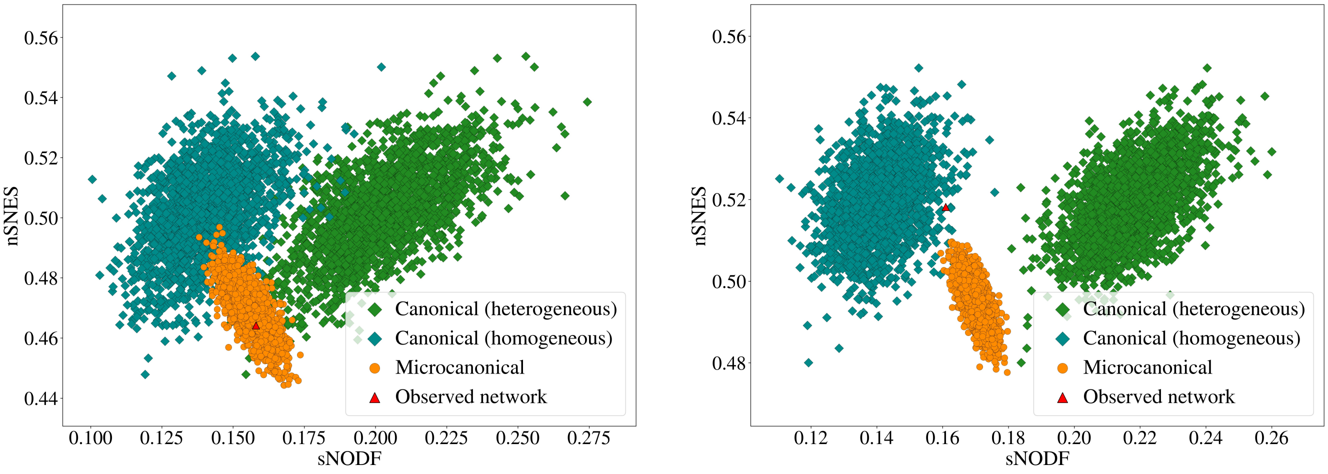

Figure D. The equivalent of Fig. 5 of the main text but for two more networks, specifically dataset 5 (left) and 1 (right). The position of the real network values with respect to the ensembles may vary.

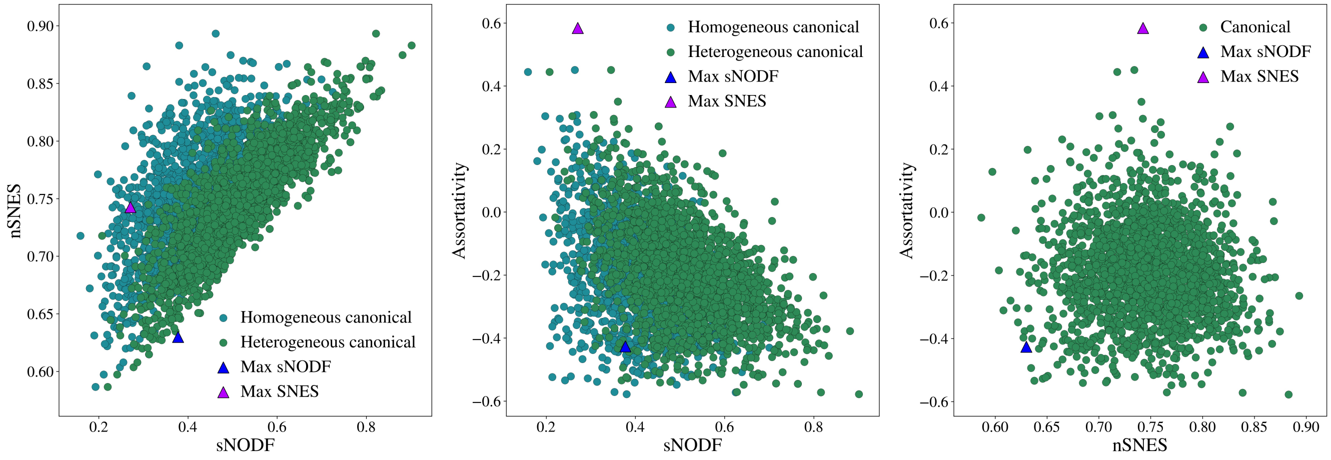

Figure E. The equivalent of Fig. 6 of the main text but for the canonical ensemble. The blue and violet realizations are the matrices of maximum sNODF and SNES of the microcanonical ensemble.

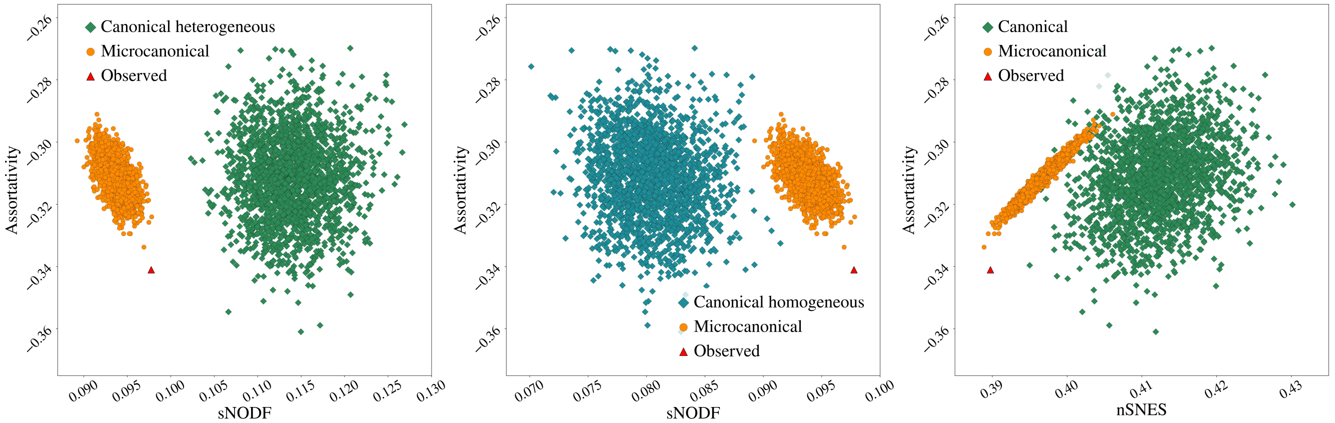

Figure F. The correlation between the nestedness measures and assortativity is hidden in the canonical ensemble. The Spearman correlation coefficients are, from top to bottom: -0.51 and -0.04 for the top figure, -0.17 and -0.51 for the middle figure, 0.97 and 0.23 for the bottom one.

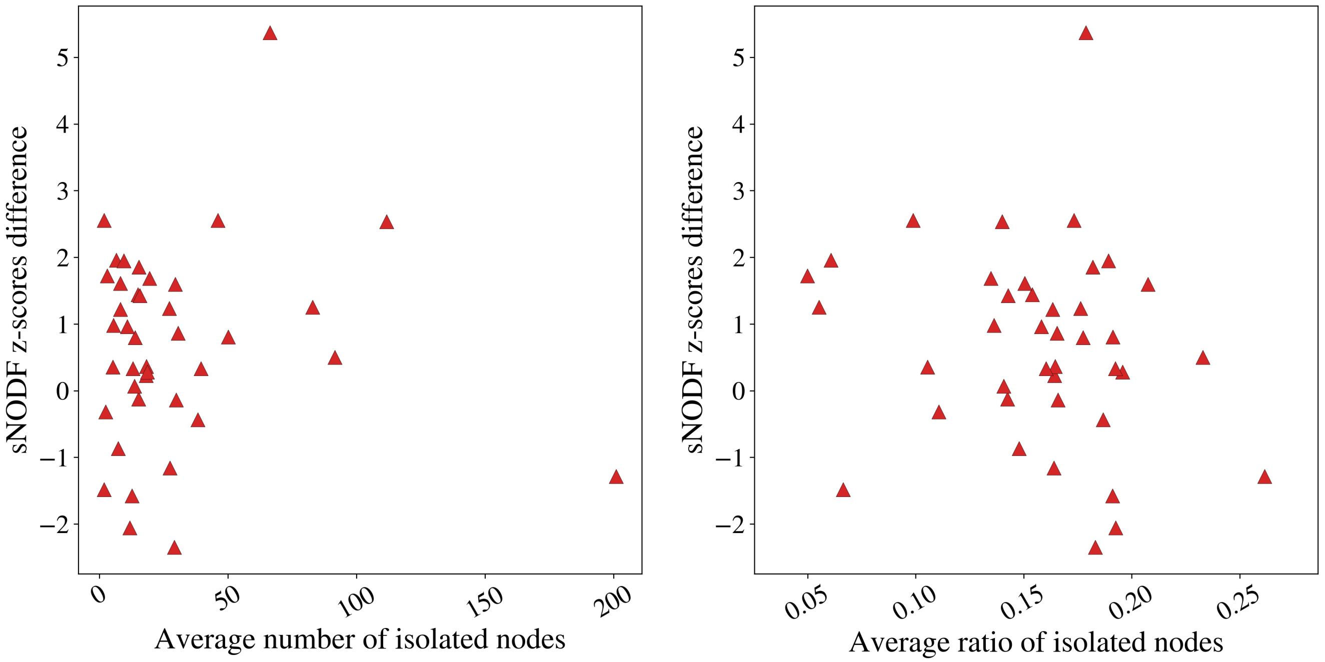

Figure G. The correlation between the number of isolated nodes in the samplings and the difference between the sNODF homogeneous canonical z-scores and the sNODF microcanonical z-scores. Neither the number of isolated nodes nor their ratio seem to be factors, with Spearman correlation coefficients of 0.02 and -0.25 respectively.

References

1. Lee, S. H. Network nestedness as generalized core-periphery structures. *Phys. Rev. E* **93**, 022306, DOI: 10.1103/PhysRevE.93.022306 (2016).
